# Antimalarial Efficacy and Safety of *Senna occidentalis* (L.) Link Root Extract in *Plasmodium berghei*-Infected BALB/c Mice

**DOI:** 10.1155/2023/8296195

**Published:** 2023-08-07

**Authors:** Simeon Mogaka, Isaac Mulei, Peninah Njoki, Kenneth Ogila, Rebecca Waihenya, Faith Onditi, Hastings Ozwara

**Affiliations:** ^1^Department of Tropical and Infectious Diseases, Institute of Primate Research, P.O. Box 24481, Karen, 00502 Nairobi, Kenya; ^2^Department of Zoology, Jomo Kenyatta University of Agriculture and Technology, P.O. Box 62000-00200 Nairobi, Kenya; ^3^Department of Veterinary Pathology, Microbiology and Parasitology, University of Nairobi, P.O. Box 29053, 00625 Nairobi, Kenya; ^4^Department of Medical Science, Technical University of Mombasa, P.O. Box 90420-80100, Mombasa, Kenya; ^5^Laboratory of Malaria Immunology and Vaccinology, National Institute of Allergy and Infectious Diseases, National Institutes of Health, Rockville, MD, USA

## Abstract

**Background:**

Emergence of *Plasmodium* resistance to antimalarial drugs presents a major drawback in efforts to control malaria. To address this problem, there is an urgent and continuous need for the development of new and effective antimalarial agents. *Senna occidentalis* (L.) link extract has exhibited *in vitro* antiplasmodial activity in many pharmacological studies. To our knowledge, data on its *in vivo* antimalarial efficacy is still very limited. A recent study demonstrated that polar extracts from the plant roots inhibit *Plasmodium berghei* proliferation in a mouse model. This study further describes the efficacy and safety of a methanolic root extract of the plant as an antimalarial agent by demonstrating its effect on hematological, biochemical, and histological parameters of *Plasmodium berghei*-infected BALB/c mice.

**Methods:**

Rane's test, a curative approach, was used to evaluate the antimalarial efficacy of *Senna occidentalis* methanolic root extract in *Plasmodium berghei*-infected BALB/c mice. The effect of the extract on both hematological and biochemical parameters was evaluated using automated analyzers. Kidney, liver, lung, spleen, and brain tissues were harvested from euthanized mice and examined for changes in organ architecture.

**Results:**

This study demonstrates that methanolic root extract of *Senna occidentalis* significantly inhibited *Plasmodium berghei* parasitemia in BALB/c mice (*p* < 0.01). Infected mice that were treated with the extract depicted a significantly low level of total leucocytes (*p* < 0.01), red blood cell distribution width (*p* < 0.01), and a significantly high hemoglobin concentration (*p* < 0.001) compared to the infected animals that were administered with the vehicle only. The infected animals that were treated with the extract exhibited a significantly low level of urea, creatinine, bilirubin, and alkaline phosphatase (*p* < 0.05), compared to the infected animals that were given the vehicle only. The level of sodium, potassium and chloride ions, lymphocytes, granulocytes, hematocrit (HCT), mean corpuscular volume (MCV), mean corpuscular hemoglobin (MCH), mean corpuscular hemoglobin concentration, total protein, albumin, aspartate aminotransferase (AST), alanine transaminase (ALT), alkaline phosphatase (ALP), total platelets, mean platelet volume (MPV), and platelet distribution width of the infected animals treated with the extract was not significantly different from those of the infected animals that were given the vehicle only (*p* > 0.05). The extract alleviated organ pathological changes in the infected mice. The extract did not induce any remarkable adverse effect on the growth, hematological, and biochemical parameters of uninfected animals (*p* > 0.05). In addition, administration of the extract did not alter the gross appearance and histological architecture of the organs, implying that the extract was well tolerated in mice.

**Conclusions:**

*Senna occidentalis* methanolic root extract exhibited good antimalarial activity against *Plasmodium berghei* and may be safe in mice.

## 1. Introduction

Malaria continues to pose significant challenges in public health, especially in resource-constrained Sub-Saharan African regions [1–3]. There were approximately 247 million malaria cases and 619000 deaths globally in 2021 [4]. Vector control and drug therapy have led to a remarkable success in the fight against malaria [2, 5]. However, the continued emergence of resistance to insecticides and to the available antimalarial drugs presents a major drawback in efforts to control the disease [3, 6, 7]. This necessitates a continued search for alternative insecticides and antimalarial drugs to combat this globally emerging threat.

Traditional medicine has shown great potential for the management of various health conditions [8] and has remained the most accessible and affordable means of treatment, especially in Sub-Saharan Africa [5, 9, 10]. Natural products constitute an important resource in both traditional and conventional medical systems throughout the world. Approximately 40% of approved drugs available in the market are either natural products or their derivatives [10, 11]. Given the long history of use by humans, herbal medicine is assumed to be safe [12]. In contrast, emerging evidence reveals safety concerns regarding the use of some of these medicinal products [8, 13]. Therefore, there is a need to scientifically ascertain the efficacy and safety of these therapies.

*Senna occidentalis* has been used in folk medicine to manage various health problems [14, 15]. The plant is used traditionally to manage malaria in Nigeria [16], Congo [17], and Kenya [18–20]. A number of studies have shown that extracts from the plant exhibit notable antiplasmodial activity *in vitro* [14, 16, 17, 21]. More recently, we demonstrated that polar extracts from the plant root possesses good antiplasmodial activity, *in vitro* and in mice [22]. On the other hand, existing reports on *S. occidentalis* safety and toxicity are inconsistent [23–31]. The variation in toxicity is influenced by various factors such as the place of origin of the plant, the season of collection, the part of the plant used, the method of extract preparation, the dose administered, and the length of “drug” exposure, all of which have been shown to affect the bioactivity of *S. occidentalis* [24, 26, 31]. As such, this study sought to further evaluate the plant for antimalarial efficacy and safety in *Plasmodium berghei*-infected BALB/c mice by characterizing the effect of methanolic root extract of the plant on hematological, biochemical, and histopathological indices.

## 2. Materials and Methods

### 2.1. Plant Collection and Extraction

*Senna occidentalis* was identified by Mr. Jonathan Ayayo, a taxonomist of the National Museums of Kenya (NMK), and a voucher specimen (38/81) was deposited at the East African Herbarium, NMK for future reference. The plant roots were collected from Migori County (0.9366^o^ S, 34.4198^o^ E), western Kenya, in the month of September in accordance with the WHO Guidelines on Good Agriculture and Collection Practices (GACP) for medicinal plants [32]. Air-dried roots were ground into powder, and absolute methanol was used to prepare the extract by maceration at room temperature for 48 h. The filtrate (Whatman No. 1 filter paper) was concentrated by rotary vaporization at 50°C and reduced pressure (BÜCHI R-200 rotary evaporator), and the extract (paste) was stored in sealed sample bottles at 4°C until needed.

### 2.2. Ethical Approval and Experimental Mice

Approval to conduct the study (ISERC/02/18) was granted by the Institutional Scientific and Ethics Review Committee of the Institute of Primate Research (IPR). Male and female inbred BALB/c mice aged 8-9 weeks were used in this study. The animals were provided by IPR rodent facility. The handling and care of the animals were in compliance with international guidelines on the care and use of laboratory animals. Besides, the Animal Research: Reporting In *Vivo* Experiments (ARRIVE) guidelines were followed in the conduct of this study.

### 2.3. Mice Infection and Treatment

*Plasmodium berghei*, strain ANKA, was sourced from the Kenya Medical Research Institute (KEMRI) and maintained in our laboratory by serial passage in mice [33] for use in this study. The parasite strain causes death in untreated mice after 7-10 days of infection, with parasitemia reaching 10-20% [5]. Factors such as the route of infection and parasite inoculum may influence the onset of mortality in *P. berghe*, ANKA-infected BALB/c mice, with deaths occurring as early as day 6 after infection [34–36]. Mice were infected, treated, and sampled as previously described [37, 38], with a few modifications. Briefly, blood was collected from *P. berghei-*infected mice and used to inoculate intraperitoneally 20 mice (1 × 10^6^*P. berghei*-infected red blood cells per animal). The mice were randomly grouped into 4 groups (A, B, C, and D) of 5 animals each. Another set of 10 uninfected mice, 5 animals each (groups E and F), was included. On day 4 postinfection, the animals in groups A and B were given 100 mg/kg and 200 mg/kg doses of the methanolic extract diluted in phosphate buffered saline (vehicle), respectively. Group C was given 1 mg/kg pyrimethamine (Sigma–Aldrich Chemie, Steinheim, Germany), while group D was administered with the vehicle only. Group E was given 200 mg/kg extract, while group F was given PBS, vehicle only. The treatment (0.2 ml per mouse) was done orally once a day for 4 consecutive days. The concentration of extract administered was based on previous dose recommendations [39–41].

### 2.4. Evaluation of Parasitemia Suppression

The effect of *S. occidentalis* methanolic root extract on *P. berghei* propagation in mice was determined as described [37]. Briefly, thin smears were prepared from tail blood on day 4 postinfection (start of treatment) and on day 8 postinfection (24 hours after the treatment regimen), fixed with absolute methanol and observed under the light microscope. The number of parasitized red blood cells (pRBCs) was determined per at least 1000 RBCs. Percentage parasitemia was computed using the formula: parasitemia (%) = (y/z)100 where *y* = number of infected RBCs and *z* = total number of RBCs counted. Parasite suppression (%) was then computed as %suppression = ((a − b)/a)100, where a = percentage parasitemia in the untreated control group and b = % parasitemia in the treated group [42].

### 2.5. Determination of Weight Changes

*Plasmodium* infection is known to cause decreased appetite which results in weight loss [1]. Similarly, studies in experimental animals have demonstrated that drug toxicity is also linked to weight loss [8]. To assess the effect of the extract on weight changes in *P. berghei*-infected and uninfected mice, the average change in body weight of the mice was assessed and compared within experimental groups on day 0 (day of infection) and day 8 postinfection (24 hours after the treatment regimen).

### 2.6. Determination of Hematological and Biochemical Parameters

The experimental mice were euthanized by carbon dioxide asphyxiation on day 8 postinfection (after an overnight fast), and whole blood was collected (cardiac puncture) in ethylenediamine tetraacetic acid (EDTA). The blood was examined using an automated hematology analyzer (CC2400Plus Cell Counter) to determine the extract impact on hematological parameters in *P. berghei*-infected and uninfected mice. Red blood cell, white blood cell, and platelet indices were recorded, and comparisons were drawn among the groups. To assess the effect of the extract on kidney and liver function, blood was collected (in tubes with clot activating factor), processed into serum, and analyzed (Humalyzer primus-200) for the levels of urea, creatinine, and electrolytes. In addition, measurements and comparisons for bilirubin, albumin, total protein, aspartate aminotransferase (AST), alanine transaminase (ALT), and alkaline phosphatase (ALP) were also made.

### 2.7. Macroscopic and Histopathological Examination of Organs

*Plasmodium* infection affects mainly the kidney, liver, spleen, lungs, and brain [43]. Moreover, the liver and kidney are very vulnerable to toxicity [10]. To assess the effect of *S. occidentalis* extract on gross pathological alterations, the organs were collected on day 8 postinfection and examined macroscopically for changes in color and size. For histopathological examination, tissue sections were prepared from the organs and examined for changes in architecture. The preparation of the organs, sectioning, and staining were done as described [43] with some modifications. Briefly, the organs were collected and preserved in 10% (*v*/*v*) solution of buffered formalin (neutral) for 7 days. Samples (5 mm thick) were dehydrated in 80, 95, and finally, 100% ethanol. The tissues were cleared with toluene, impregnated, and embedded in paraffin wax. From the tissues, 6 *μ*m sections (Rotary Microtome, Leitz 1512, Germany) were made, mounted on glass slides, dewaxed, cleared with xylene, rehydrated with a descending series of ethanol (100, 95, and 80% ethanol), and stained with hematoxylin and eosin solution. The slides were cleaned in 2 changes of xylene, cover-slipped, and mounted using dibutylphthalate polystyrene xylene (DPX) mountant (Sigma) and let to dry. The tissue sections were then examined for changes in architecture, and comparisons were drawn among the experimental groups.

### 2.8. Statistical Analysis

The data were recorded as means ± standard error of the means (M ± SEM). *In vivo* parasite suppression, hematological, and biochemical parameters were analyzed through an ordinary one-way ANOVA followed by Tukey's multiple comparison test. Changes in weight between day 0 (infection day) and day 8 postinfection were analyzed by student's *t*-test. GraphPad Prism (Version 7.00, California, USA) was used for the analyses. Differences were considered significant when *p* < 0.05.

## 3. Results

### 3.1. Effect of *Senna occidentalis* Methanolic Root Extract on *Plasmodium berghei* Propagation in Mice

The effect of a methanolic root extract of *S. occidentalis* on *P. berghei* propagation was determined by assessing parasitemia levels in the infected mice on day 8 postinfection (24 hours after treatment with the extract) relative to the infected but untreated mice. Our study demonstrated that mice treated with 100 and 200 mg/kg body weight of the extract exhibited a 2-fold decrease in *P. berghei* parasitemia levels when compared to the control group. When compared to the control group, the parasitemia suppression (54%) was significant (*p* < 0.01) in the extract-treated mice. There was no discernible difference in parasitemia suppression (*p* > 0.05) between the two extract doses, suggesting that a dose of 100 mg/kg body weight of the extract is as effective as that of 200 mg/kg body weight. On the other hand, the pyrimethamine-treated group effectively eliminated the parasites, showing approximately 100% *P. berghei* parasitemia suppression, as illustrated in Table 1. Antimalarial agents with good activity have been shown to have parasite percentage suppression ≥50 at doses of 100 to 250 mg/kg body weight per day [44]. As such, this study shows that *S. occidentalis* methanolic root extract demonstrated notably good antimalarial activity during the *in vivo* testing. Therefore, the methanolic root extract was further assessed for impact on weight changes and hematological, biochemical, and histopathological changes in the mouse malaria model.

### 3.2. Effect of *Senna occidentalis* Methanolic Root Extract on Weight Changes in Mice

To determine the effect of methanolic extract of *S. occidentalis* roots on weight changes in *P. berghei* infected and uninfected mice, animal weights were recorded and comparisons drawn between the day of infection (day 0) and day 8 postinfection (24 hours after administering the final dose of treatment), as illustrated in Table 2. Mice in group D (infected but not treated) and the extract-treated mice lost weight significantly (*p* < 0.05) during the experimental period. On the other hand, pyrimethamine-treated animals gained weight during the experimental period although this change was not significant when compared to the untreated animals (*p* > 0.05). The difference in weight loss between 100 and 200 mg/kg doses of the extract was not significant (*p* > 0.05). Likewise, there was no significant change in weight between extract- and vehicle-administered uninfected mice (*p* > 0.05). Though the extract-treated mice lost weight significantly during the infection period, a comparison of weight loss between extract-treated and untreated mice shows that the extracts minimized weight loss resulting from *P. berghei* infection by at least 3.67%. Further, results from the uninfected mice show that the animals tolerated the extract dose.

### 3.3. Effect of *Senna occidentalis* Methanolic Root Extract on Hematological Parameters in Mice

Blood plays an important role as a marker of physiological and pathological changes in humans and other animals [10]. The change in hematological parameters during malarial infection is a well-documented phenomenon [45]. Parameters such as white blood cell count, platelet count, red blood cell count, and hemoglobin concentration are important indicators of antimalarial drug efficacy [38]. To evaluate the effect of *S. occidentalis* methanolic root extract on hematological indices in *P. berghei*-infected and uninfected mice, the animals were administered with the extract, and blood samples were collected and analyzed for leucocyte, erythrocyte, and platelet indices.

#### 3.3.1. Effect of the Extract on White Blood Cell Indices

Total leukocytes significantly increased in BALB/c mice infected with *P. berghei* (*p* < 0.05) compared with the uninfected animals. The composition of lymphocytes and granulocytes, however, was not significantly changed by the infection (*p* > 0.05). Infected mice treated with the extract and the standard drug pyrimethamine experienced a significant drop in their total white blood cell count (*p* < 0.01) compared with the infected animals that were administered with vehicle only. Neither the extract nor pyrimethamine treatment significantly altered the lymphocyte and granulocyte (*p* > 0.05) composition. Mice administered with 200 mg/kg extract had a significantly lower total leucocyte count compared to the 100 mg/kg extract administered group (*p* < 0.01). However, there was no significant difference in lymphocyte and granulocyte population between mice administered the two extract doses (*p* > 0.05). The administration of the extract to uninfected mice did not induce any remarkable changes in leucocyte indices (*p* > 0.05). These findings affirm that treatment with the extract reduced the negative effect of *P. berghei* infection on the total leucocyte count. Absence of leukocytosis or leukocytopenia in the uninfected animals given the extract demonstrates that differences in parasitemia burden may be responsible for the observed changes in leucocyte indices in infected mice.  Table 3 shows the effect of *Senna occidentalis* root extract on white blood cell indices of *P. berghei*-infected and uninfected BALB/c mice compared with controls.

#### 3.3.2. Effect of the Extract on Red Blood Cell Indices

*Plasmodium berghei*-infected mice exhibited decreased levels of total red blood cell count, hemoglobin, percentage hematocrit, and mean corpuscular hemoglobin concentration, which were not significant (*p* > 0.05) compared with uninfected animals, as illustrated in Table 4. Red blood cell distribution width (RDW-SD) was insignificantly increased (*p* > 0.05) in the infected animals. Infected mice treated with the extract had insignificantly (*p* > 0.05) elevated total RBC count and hematocrit percentage compared with the animals given the vehicle only. Mice treated with the extract (200 mg/kg) and pyrimethamine exhibited a significantly higher hemoglobin concentration compared to the untreated infected mice (*p* < 0.001). While the total red blood cell count and hematocrit level were both greater in the pyrimethamine-treated group compared with the vehicle-only administered animals, the observed difference was not significantly different (*p* > 0.05). In comparison to the infected group that received the vehicle only, the extract (200 mg/kg) and pyrimethamine-treated mice displayed a significantly low RBC distribution width (*p* < 0.01). With the exception of hemoglobin concentration (*p* < 0.05) and RDW-SD (*p* < 0.01), there was no significant difference between the effects of 100 and 200 mg/kg extract on red blood cell indices (*p* > 0.05). Uninfected mice that received the extracts did not display any noticeable change in their RBC indices (*p* > 0.05). These findings imply that using the extract as an antimalarial agent reduces the negative effect of *P. berghei* infection in BALB/c mice on some red blood cell indices. These findings further show that the animals tolerated the extract for it did not stimulate or suppress erythropoiesis in the uninfected mice.

#### 3.3.3. Effect of the Extract on Platelet Indices

Infection with *P. berghei* caused a significant reduction in platelet count in BALB/c mice (*p* < 0.05) but had no discernible effect on mean platelet volume or platelet distribution width compared with uninfected animals (*p* > 0.05). The total platelet count, mean platelet volume, and platelet distribution width did not change significantly after the extract and pyrimethamine treatment of the infected mice compared to the vehicle-only administered animals (*p* > 0.05). Similarly, the extract had no notable impact on the platelet indices of uninfected mice (*p* > 0.05).  Table 5 shows the effect of the *S. occidentalis* extract on platelet indices. The results demonstrate that the extract did not have any remarkable impact on the platelet indices of both infected and uninfected mice.

### 3.4. Effect of *Senna occidentalis* Methanolic Root Extract on Kidney and Liver Function Indicators in *Plasmodium berghei*-Infected and Uninfected BALB/c Mice

Toxin filtration and metabolism occur in the kidney and liver, respectively. As such the two organs are extremely vulnerable to toxicity [10]. In addition, these organs are very prone to injury resulting from malaria infection [10, 43]. In this study, methanolic root extract of *S. occidentalis* was administered orally to both *P. berghei*-infected and uninfected mice to assess its impact on kidney and liver function parameters.

#### 3.4.1. Effect of Root Extract on Kidney Function Parameters

*Plasmodium berghei* infection in mice did not significantly alter serum electrolytes (*p* > 0.05) in the animals compared with uninfected animals. However, the infection significantly increased serum urea and creatinine levels in the mice (*p* < 0.001). Treatment with the extract and control drug, pyrimethamine, had no significant impact on electrolyte levels of the infected mice (*p* > 0.05) compared with the vehicle-only administered animals. In contrast, the infected mice treated with the extract and control drug exhibited significantly reduced urea and creatinine levels (*p* < 0.01) compared with the vehicle-only administered animals. There was no significant difference between the effect of 100 and 200 mg/kg of extract on kidney function parameters (*p* > 0.05) in the infected animals. Administration of extract to uninfected mice had no marked impact on all kidney function parameters investigated (*p* > 0.05) compared with vehicle-only administered animals. The effect of the extract on renal function parameters is illustrated in Table 6. These observations affirm that *S. occidentalis* methanolic root extract lessened the negative effect of *P. berghei* infection on urea and creatinine levels in BALB/c mice. In addition, the results show and that the extract is well tolerated in this mouse model at the doses investigated.

#### 3.4.2. Effect of the Extract on Liver Function Parameters

The levels of total bilirubin, direct bilirubin, alanine transaminase (ALT), and alkaline phosphatase (ALP) were significantly elevated in the *P. berghei*-infected mice (*p* < 0.05) compared with the uninfected animals. The level of total protein was also raised significantly (*p* < 0.05) in the infected animals. Albumin and aspartate aminotransferase (AST) levels were similarly elevated in mice but not significantly (*p* > 0.05). An increase in AST/ALT ratio correlates with diminishing liver function [46]. In this study, the AST ratios were 2.08, 2.05, 1.76, 2.16, 1.47, and 1.34 for animals in experimental groups A, B, C, D, E, and F, respectively. Mice that were infected with the *P. berghei* parasites and treated with the extract or standard drug, pyrimethamine, had significantly lower levels of bilirubin and ALP compared to the infected mice that were given the vehicle only (*p* < 0.05). The extract had no marked impact on total protein and albumin levels in the infected animals (*p* > 0.05). The standard drug, pyrimethamine, significantly lowered the total protein levels (*p* < 0.05) in the infected animals. The infected mice that were given the extract and control drug exhibited insignificantly reduced levels of AST and ALT (*p* > 0.05). Similarly, the impact of 100 and 200 mg/kg extract on liver function parameters did not differ significantly (*p* > 0.05), except for the bilirubin level, which was significantly higher (*p* < 0.05) in infected animals that were administered the latter dose. The administration of the extract to uninfected mice had no distinguishable impact on liver function parameters (*p* > 0.05). The effect of the extract on hepatic function is displayed in Table 7. Low levels of ALP, bilirubin, and AST/ALT ratio in the infected mice that were treated with the extract are indicative of reduced liver injury resulting from *Plasmodium* infection. Further, the results demonstrate that the animals tolerated the extract doses administered here.

### 3.5. Effects of *Senna occidentalis* Methanolic Root Extract on Organ Pathological Changes in Mice

Organs (kidney, liver, spleen, lungs, and brain) harvested from *P. berghei*-infected and uninfected mice were examined macroscopically for changes in color and size to determine the effect of the administered *S. occidentalis* methanolic root extract. The kidney, liver, spleen, lungs, and brain of the mice treated with extract or pyrimethamine appeared normal. The organs were similarly normal for uninfected mice that received the extract. Infected animals that received the vehicle only had normal kidneys, dark and enlarged livers and spleens, dark lungs, and pale brains.

**Figure 1:**
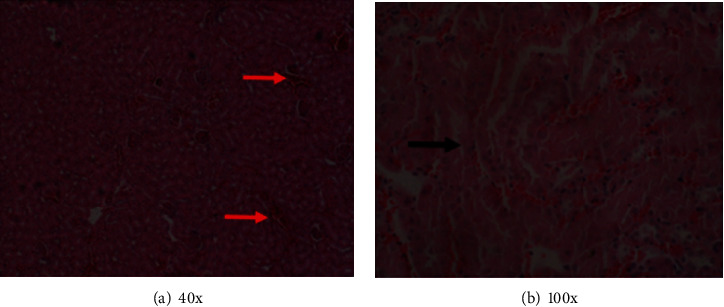
(a) Hemorrhages in the kidney (red arrows). (b) Tubular degeneration (black arrow) and hemorrhages.

**Figure 2:**
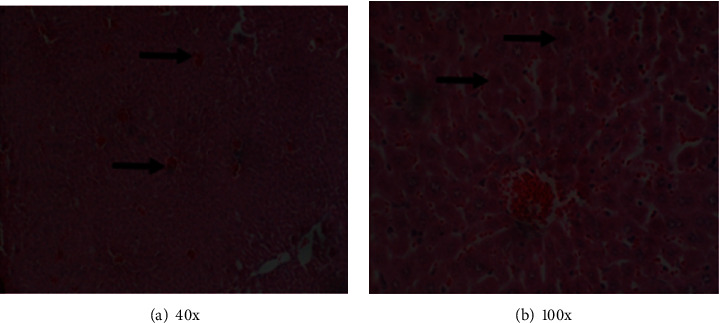
(a) Congestion of hepatic blood vessels. (b) Hepatocyte degeneration without a visible nucleus (black arrows) and hemorrhages.

**Figure 3:**
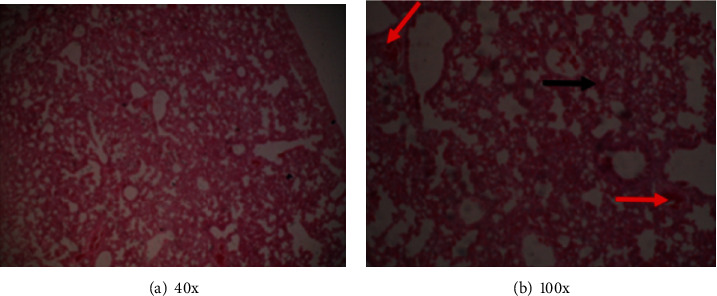
(a) Interstitial cellular infiltration, collapsed alveoli, and emphysema while (b) interstitial hemorrhages (black arrow) and congested blood vessels (red arrows).

In the histological examination of the organs, animals in groups A, B, C, E, and F did not exhibit histopathological changes suggestive of organ damage. However, animals in group D, which were infected and received the vehicle, displayed severe tissue pathology changes in the organs that were examined. Tubular and glomerular hemorrhages with tubular degeneration were in the kidneys of the animals (Figures 1(a) and 1(b)). Hemorrhages, hepatocyte degeneration, loss of hepatic cord pattern, and general loss of lobular architecture were evident in the liver (Figures 2(a) and 2(b)). There was interstitial cellular infiltration, collapsed alveoli, emphysema, interstitial hemorrhages, and congested blood vessels in the lungs of these animals (Figures 3(a) and 3(b)). Other observations included the proliferation of lymphocytes with hyperplasia of germinal centers in the spleen and congestion of the cerebral vasculature. These results suggest that the extract and the control drug pyrimethamine minimized pathology resulting from *P. berghei* infection in BALB/c mice. Additionally, these findings imply that there was no detectable organ damage caused by the extract, implying that the short-term use of the extract doses used here was safe and well tolerated by the animals.

## 4. Discussion

The current study assessed the efficacy and safety of a methanolic root extract of *S. occidentalis* as a cure for *P. berghei* ANKA infection in BALB/c mice. The treatment of the infected mice with the extract inhibited parasitemia in mice, thereby modulating some hematological and biochemical changes in the animals. In addition, the extract treatment alleviated organ damage resulting from murine malaria parasite infection. These observations corroborate previous findings where extracts from the plant species were reported to exhibit antiplasmodial activity *in vitro* [14, 16, 17, 21] and *in vivo* [41].

Reduced water and feed intake, coupled with loss of body weight, are indicators of poor health status in animals [8]. One of the hallmarks of malaria infection is loss of body weight [47]. An ideal antimalarial drug should be able to prevent severe weight loss during infection [6]. In this study, mice infected with *P. berghei* experienced a significant weight loss. Weight loss during malaria infection may be attributed to a reduction in feed intake and metabolism accompanied with hypoglycemia [1, 47]. Treatment of *P. berghei*-infected mice with *S. occidentalis* methanolic root extract minimized weight loss, though not remarkably, in the current study. Other studies have reported restricted weight loss in murine malaria-infected mice following treatment with plant extracts with antimalarial properties [6, 48–50]. In the uninfected mice, this study demonstrated that treatment with *S. occidentalis* root extract did not affect weight gain. This observation concurs with the findings of a previous study where leaf and stem extracts of *Cassia occidentalis* (synonym: *S. occidentalis*) were found not to induce weight changes in Wistar rats [29].

Hematological indices play a well-established role in predicting normal biological processes and responses to pharmacological substances during therapy or adverse effects of foreign compounds [45, 51]. Infection with malaria parasites alters the host's hematological parameters [45]. On the other hand, parameters such as white blood cell count, platelet count, red blood cell count, and hemoglobin concentration are important indicators of antimalarial drug efficacy [38]. In the current study, *P. berghei* infection in mice deviated various hematological indices from normal status. Using *S. occidentalis* methanolic root extract to treat the infected mice ameliorated some of these parameters, demonstrating its effectiveness in reducing malaria pathogenesis.

*Plasmodium berghei* infection was associated with an elevation of host white blood cell count in the current study. This observation is consistent with findings of previous studies of *P. falciparum* in humans [52, 53]. However, a low to normal leucocyte count is more common during *Plasmodium* infection [45, 54]. Reduction in leucocyte count in the infected animals result from the sequestration in the spleen, which in turn limits their peripheral circulation [45, 55]. In the current study, the *P. berghei*-infected animals that were treated with the extract and standard drug pyrimethamine demonstrated a reduction in their white blood cell count. This reduction in leucocyte number was in tandem with the decreased parasite load. The occurrence of leucocytosis or leucopenia during *Plasmodium* infection depends on the parasite burden with leucocytosis positively correlating with a high parasite density [45]. Leucocytosis or leucocytopenia may also result from the malfunctioning of myeloid and lymphoid stem cells. Factors that alter the production of colony-stimulating factors and interleukins would affect production of leucocytes [51]. In this study, *S. occidentalis* extract had no impact on the production of leukocytes in uninfected mice. On the contrary, it was previously reported that a stem bark extract from *S. occidentalis* promoted white blood cell production in rabbits [56]. The observed variation in leucocyte synthesis activity between the two studies may be explained by differences in plant part and dosage used [57, 58].

Anemia resulting from reduced erythrocyte count is the most common complication of malaria [38]. As expected in this study, *P. berghei*-infected mice exhibited a reduced erythrocyte count. The reduction in red blood cell count could be attributed to the phagocytosis of erythrocytes as well as the inhibition of erythropoiesis by the accumulated malaria pigment, hemozoin [1, 59, 60]. The sequestration and rosetting of the infected cells in various organs may also contribute to this reduction [45]. These events may lead to deviations in some erythrocyte indices of the infected host. During the erythrocytic stage, malaria parasites degrade host hemoglobin in order to obtain amino acids for their own use [61]. This may account for the observed decrease in hemoglobin concentration during *Plasmodium* infection in the current study. The potential of an antimalarial drug candidate to restore normal hematological parameters following infection is highly sought-after [1]. The significantly high hemoglobin concentration and the reduced red blood cell distribution width in the extract-treated group compared to the untreated group are desirable, even though the plant extract used here did not cause a notable change in many of the red blood cell indices of infected mice. Animal tissues require oxygen for cellular respiration, which is transported to the tissues by hemoglobin. It also carries carbon dioxide away from the tissues [38].

The *Plasmodium* infection led to a significant reduction in platelet count in the current study. A reduction in platelet count during malaria infection has similarly been observed in other studies [52]. Thrombocytopenia, as observed in the current study, may have resulted from the engulfment of *Plasmodium-*infected erythrocytes by platelets, thus marking them for destruction by phagocytes [38, 45, 62]. It may also have occurred due to disseminated intravascular coagulation (DIC) following the infection [52]. In the present study, treatment with *S. occidentalis* methanolic root extract did not accrue any remarkable beneficial or detrimental effects on the platelet indices of both infected and uninfected animals. Similar to the results of the current study, *C. occidentalis* leaf and stem extracts failed to alter the hematological profiles of uninfected Wistar rats, in a separate study [29].

An increase in protein in urine and elevated levels of urea, creatinine, and electrolyte imbalances are all indicators of a dysfunctional kidney [51, 63]. In this study, *P. berghei*-infected mice exhibited an increase in urea and creatinine levels. Other studies have similarly reported elevated renal function parameters in malaria-infected individuals [64]. Similar to an observation made previously regarding infection of humans with *P. falciparum* [65], *P. berghei* infection did not alter the level of serum electrolytes in the present study. Treatment of infected mice with methanolic root extract of *S. occidentalis* and pyrimethamine ameliorated some renal function parameters in the animals. The improvement in renal function parameters was in tandem with reduced parasite burden. Similar to the observations herein, high parasite density has been associated with diminished renal function during *Plasmodium* infection [64].

Liver injury is characterized by an increase in the blood levels of aspartate aminotransferase (AST), alanine transaminase (ALT), and alkaline phosphatase (ALP) [10, 51, 66, 67]. In the current study, total protein, bilirubin, ALT, and ALP were significantly elevated in *P. berghei*-infected mice, an indication of decreased liver function. This observation concurs with previous reports regarding *P. falciparum* infection in human [65, 68]. The AST/ALT ratio was at least 1.4-fold higher in the untreated *P. berghei*-infected mice compared with the uninfected animals. Elevation of the AST/ALT ratio in infected mice could be explained by hemolysis of RBCs due to the infection since erythrocytes contain AST [46]. Erythrocyte hemolysis leads to the generation of hemozoin, which plays an important role in oxidative damage of the liver [46]. In the present study, *S. occidentalis* methanolic root extract improved the outcome of *P. berghei* infection on bilirubin and ALP levels in mice, an observation that affirms its antimalarial potency. The extract did not significantly alter liver function parameters in uninfected mice. This observation concurs with previous finding involving leaf and stem extracts of the plant in which the extracts were found not to alter biochemical profiles in Wistar rats [29]. Contrary to these results, a leaf extract from the plant species significantly altered the liver function parameters in uninfected Wister rats in a previous study [27], an indication of toxicity.

There are emerging concerns regarding the safety of phytomedicines [8, 13]. In the present study, the uninfected mice that received the methanolic extract from *S. occidentalis* roots had no appreciable unfavorable changes in their weight, hematological, biochemical, and histological parameters. Our findings concur with previous observations on the toxicity of *S. occidentalis* stem and leaf extracts [23, 29, 30]. Contrary to the current findings, *S. occidentalis* was shown to significantly alter some investigated parameters in previous studies [24–28, 31]. The observed variation in these studies may be explained by differences in the plant's place of origin, the season of collection, the part used, the method of preparation, the dose administered, and the length of exposure [24, 26, 31]. The current study shows that the plant root extract may be safe at the tested concentrations because there were no noticeable harmful effects in the uninfected mice that were given the extract. However, it will be crucial to investigate the safety of the extract in the context of long-term use as an antimalarial remedy.

The liver, kidney, brain, lungs, and spleen constitute the major organs affected during severe malaria infection [43, 69]. In the current study, the infection of mice by *P. berghei* resulted in severe tissue pathology in the organs investigated. Infected mice that were treated with the extract exhibited normal tissue architecture, implying that the treatment attenuated the effect of the infection on the organs. Additionally, administration of the extract to uninfected mice did not result in any pathological changes to the organs, showing that the extract was well tolerated by the animals at the doses administered. Similar to the current findings, a toxicological evaluation of this plant's stem and leaf extracts in Wistar rats did not alter the macroscopical and microscopical appearance of organs, in a previous study [29]. On the other hand, *S. occidentalis* triturated seeds were found to cause organ damage in Wistar rats [24]. The current results on organ pathology affirm further the antimalarial potency and safety of a methanolic extract of *S. occidentalis* roots as observed in the antiplasmodial, hematological, and biochemical investigations herein. Future studies will focus on assessing the prophylactic potency of the extract against malaria parasites and the evaluation of other parts of the plant for *in vivo* antimalarial activity. The evaluation of the effect of chronic administration of the plant extract for treating malaria is also desired.

## 5. Limitations of the Study

In this study, we investigated the curative potency and safety of the methanolic extract of *S. occidentalis* roots in *P. berghei-*infected mice. We did not evaluate the prophylactic potency of the plant root extract against malaria parasites. Furthermore, we did not investigate the effect of a prolonged administration of the extract on the safety profiles of the mice.

## 6. Conclusions

The findings here demonstrated that methanolic root extract from *S. occidentalis* possesses good antimalarial activity against *P. berghei*-infected BALB/c mice and may be safe. In particular, *S. occidentalis* root extract modulated *P. berghei* parasitemia in mice, thus alleviating pathological changes in the infected animals. The extract did not induce adverse effects on the growth, hematological, and biochemical parameters of uninfected animals. In addition, the extract did not alter the gross appearance and histological architecture of the kidney and the liver, suggesting that the extract may be well tolerated in BALB/c mice. However, the effect of chronic administration of the extract is yet to be elucidated. This study supports the acclaimed ethnomedical use of *S. occidentalis* roots by some communities in Kenya as an antimalarial therapy and positions the plant root as a potential source of new antimalarial principles.

## Figures and Tables

**Table 1:** *Plasmodium berghei* parasitemia and parasite suppression levels (%) in BALB/c mice following treatment with *Senna occidentalis* methanolic root extract.

Treatment type	Parasitemia (%) on day 4 postinfection	Parasitemia (%) on day 8 postinfection	Suppression (%) on day 8 postinfection
A	4.81 ± 0.42	22.96 ± 4.51	54.15
B	4.88 ± 0.29	22.87 ± 5.53	54.33
C	5.15 ± 0.19	0.17 ± 0.00	99.67
D	5.23 ± 0.31	50.08 ± 3.41	0

A: infected and given methanolic extract (100 mg/kg); B: infected and given methanolic extract (200 mg/kg); C: infected and given pyrimethamine; D: infected and given PBS (vehicle).

**Table 2:** Weight changes in *Plasmodium berghei* infected and uninfected BALB/c mice administered with *Senna occidentalis* methanolic root extract.

Experimental groups	Weight (g) day 0 (infection day)	Weight (g) on day 4 postinfection	Weight (g) on day (8 postinfection)	Change in body weight (g)	Percentage change in weight
A	27.21 ± 0.32	27.16 ± 0.65	25.23 ± 0.44 (0.01^p^)	-1.98	7.28
B	28.4 ± 0.51	28.21 ± 0.34	26.20 ± 0.30 (0.01^p^)	-2.20	7.75
C	27.73 ± 0.57	27.69 ± 0.86	28.65 ± 0.92 (0.42^p^)	+0.92	3.32
D	27.75 ± 0.48	27.63 ± 0.37	24.50 ± 0.76 (0.01^p^)	-3.25	11.71
E	28.12 ± 0.82	28.85 ± 0.66	29.45 ± 0.61 (0.23^p^)	+1.33	4.72
F	27.84 ± 0.72	28.53 ± 0.71	29.13 ± 0.83 (0.27^p^)	+1.29	4.63

A: infected and given methanolic extract (100 mg/kg); B: infected and given methanolic extract (200 mg/kg); C: infected and given pyrimethamine; D: infected and given PBS; E: not infected but given extract (200 mg/kg); F: not infected but given PBS; ^p^*p* value; -:decrease; +:increase.

**Table 3:** White blood cell indices in *Plasmodium berghei*-infected and uninfected BALB/c mice administered with *Senna occidentalis* methanolic root extract.

White blood cell parameters	Experimental groups					
	A	B	C	D	E	F
WBC (×10^9^/l)	15.85 ± 2.25^(p^^∗^^)^	6.55 ± 1.15^(p^^∗^^)^	3.45 ± 0.65^(p^^∗^^)^	28.85 ± 1.65	8.2 ± 0.8	5.95 ± 0.75
LYM (%)	88.9 ± 0.1	87.05 ± 1.55	92.9 ± 0.5	81.1 ± 5.6	84.1 ± 0.6	86.5 ± 3.05
GRAN (%)	4.75 ± 0.15	7.5 ± 1.8	3.05 ± 0.85	8.9 ± 2.1	7.35 ± 0.65	6.3 ± 2.3

^(p^^∗^^)^Statistically significant change; A: infected and given methanolic extract (100 mg/kg); B: infected and given methanolic extract (200 mg/kg); C: infected and given pyrimethamine; D: infected and given PBS; E: not infected but given extract (200 mg/kg); F: not infected but given PBS.

**Table 4:** Red blood cell indices in *Plasmodium berghei*-infected and uninfected BALB/c mice administered with *Senna occidentalis* methanolic root extract.

Red blood cell indices	Experimental groups					
	A	B	C	D	E	F
RBCs (10^12^/l)	5.56 ± 1.21	6.92 ± .11	7.15 ± 0.34	5.48 ± 0.67	7.59 ± 0.28	7.55 ± 0.05
HGB (g/dl)	10.45 ± 0.65	12.65 ± 0.25^(p^^∗^^)^	12.9 ± 0.3^(p^^∗^^)^	9.3 ± 0.9	13.3 ± 0.3	13.4 ± 0
HCT (%)	38.1 ± 3	38.7 ± 0.65	41.5 ± 1.8	33.65 ± 3.45	44.9 ± 2.7	43.4 ± 0.3
MCV (fl)	70.7 ± 10	58.1 ± 0.35	58.1 ± 0.2	61.55 ± 1.25	59.1 ± 1.4	57.5 ± 0.8
MCH (pg)	19.4 ± 3	14.2 ± 3.5	18.1 ± 0.4	17.05 ± 0.45	17.5 ± 0.3	17.75 ± 0.15
MCHC (g/dl)	27.45 ± 0.35	24.45 ± 8.13	31.1 ± 0.5	27.65 ± 0.15	29.7 ± 1.2	30.8 ± 0.2
RDW-SD (fl)	27.35 ± 4.65	21 ± 0^(p^^∗^^)^	20.75 ± 0.65^(p^^∗^^)^	30.75 ± 2.05	21.5 ± 0.9	21.1 ± 0.5

^(p^^∗^^)^Statistically significant change; A: infected and given methanolic extract (100 mg/kg); B: infected and given methanolic extract (200 mg/kg); C: infected and given pyrimethamine; D: infected and given PBS; E: not infected but given extract (200 mg/kg); F: not infected but given PBS.

**Table 5:** Platelet indices in *Plasmodium berghei*-infected and uninfected BALB/c mice administered with *Senna occidentalis* methanolic root extract.

Platelet indices	Experimental groups					
	A	B	C	D	E	F
PLT (×10^9^/l)	338.5 ± 9.5	335 ± 17.5	375.5 ± 17.5	341 ± 22	419 ± 33	451.5 ± 31.5
MPV (ft)	6.55 ± 0.05	7.7 ± 0.5	7.25 ± 1.15	7.8 ± 0.3	6.75 ± 0.05	6.2 ± 0.1
PDW (%)	8.6 ± 0.2	9.05 ± 1.05	9.5 ± 0.3	8.25 ± 0.15	9.55 ± 0.25	8.3 ± 0

A: infected and given methanolic extract (100 mg/kg); B: infected and given methanolic extract (200 mg/kg); C: infected and given pyrimethamine; D: infected and given PBS; E: not infected but given extract (200 mg/kg); F: not infected but given PBS.

**Table 6:** Kidney function indices in *P. berghei*-infected and uninfected BALB/c mice administered *Senna occidentalis* methanolic root extract.

Kidney function parameters	Experimental groups					
	A	B	C	D	E	F
Sodium (mmol/L)	156.5 ± 2.5	160.3 ± 1.5	159 ± 2	153.5 ± 0.5	155 ± 1	153 ± 2
Potassium (mmol/L)	9.95 ± 0.75	12.9 ± 1.45	10.35 ± 0.15	12.35 ± 2.42	10.9 ± 0.3	10.65 ± 2.05
Chloride (mmol/L)	106 ± 3	110 ± 2	110 ± 5	109.5 ± 0.5	112 ± 1	105 ± 2
Urea (mmol/L)	2.9 ± 0.2^(p^^∗^^)^	3.45 ± 0.15	2.9 ± 0.1^(p^^∗^^)^	3.9 ± 0.2	2.35 ± 0.25	2.1 ± 0.2
Creatinine (Umol/L)	55.8 ± 2.5^(p^^∗^^)^	53.75 ± 2.45^(p^^∗^^)^	46.4 ± 2^(p^^∗^^)^	74.05 ± 2.45	44.65 ± 1.15	41.25 ± 1.25

^(p^^∗^^)^Statistically significant change; A: infected and given methanolic extract (100 mg/kg); B: infected and given methanolic extract (200 mg/kg); C: infected and given pyrimethamine; D: infected and given PBS; E: not infected but given extract (200 mg/kg); F: not infected but given PBS.

**Table 7:** Liver function indices in *berghei*-infected and uninfected BALB/c mice administered with *Senna occidentalis* methanolic root extract.

Liver function parameters	Experimental groups					
	A	B	C	D	E	F
Total bilirubin (Umol/L)	5.7 ± 0.1^(p^^∗^^)^	6.55 ± 0.25^(p^^∗^^)^	5.4 ± 0.3^(p^^∗^^)^	10.75 ± 0.25	2.4 ± 0.1	2.15 ± 0.15
Direct bilirubin (Umol/L)	2.4 ± 0.2^(p^^∗^^)^	3.1 ± 0.1^(p^^∗^^)^	2.15 ± 0.15^(p^^∗^^)^	5.15 ± 0.25	1.25 ± 0.25	1.1 ± 0.2
Total proteins (G/L)	70.2 ± 1.1	71.3 ± 0.4	66.75 ± 1.75	71.65 ± 1.65	64.85 ± 1.65	63.25 ± 1.35
Albumin (G/L)	19.8 ± 1.4	18.65 ± 4.13	19.1 ± 0.3	17.85 ± 1.85	22.3 ± 1.7	19.05 ± 1.05
AST (U/L)	227 ± 4	224.25 ± 2.25	190.65 ± 7.35	251.4 ± 4.4	138.25 ± 3.25	130.5 ± 2.5
ALT (U/L)	109.85 ± 1.15	109.4 ± 1	108.3 ± 2.7	116.75 ± 2.95	94.35 ± 0.85	97.9 ± 2.1
ALP (U/L)	256.65 ± 6.95^(p^^∗^^)^	282.4 ± 11.2^(p^^∗^^)^	272.1 ± 2.9^(p^^∗^^)^	342 ± 6.8	203.95 ± 5.85	207.55 ± 3.83

^(p^^∗^^)^Statistically significant change; A: infected and given methanolic extract (100 mg/kg); B: infected and given methanolic extract (200 mg/kg); C: infected and given pyrimethamine; D: infected and given PBS; E: not infected but given extract (200 mg/kg); F: not infected but given PBS.

## Data Availability

Data supporting the conclusions of this study are included within the article. Raw data can be availed by the corresponding author on request.
